# Prospective Trial on the Pharmacokinetics of Clopidogrel in Hemodialysis Patients

**DOI:** 10.1016/j.ekir.2024.07.029

**Published:** 2024-07-31

**Authors:** Juergen Grafeneder, Wisse van Os, Iris K. Minichmayr, Katarina D. Kovacevic Miljevic, Birgit Reiter, Marcus D. Säemann, Veronika Machold-Fabrizii, Amro Ahmed, Paul Spechtl, Haris Omic, Raute Sunder-Plaßmann, Bernd Jilma, Christian Schoergenhofer, Farsad Eskandary

**Affiliations:** 1 Department of Clinical Pharmacology, Medical University of Vienna, Vienna, Austria; 2 Department of Emergency Medicine, Medical University of Vienna, Vienna, Austria; 3 Department of Laboratory Medicine, Medical University of Vienna, Vienna, Austria; 4 6th Medical Department, Nephrology and Dialysis, Clinic Ottakring, Vienna, Austria; 5 3rd Medical Department, Cardiology and Intensive Care Medicine, Clinic Ottakring, Vienna, Austria; 6 Department of Nephrology and Dialysis, Division of Medicine III, Medical University of Vienna, Vienna, Austria

**Keywords:** cardiovascular risk, clopidogrel, cytochrome P450, hemodialysis, pharmacokinetics, platelet aggregation

## Abstract

**Introduction:**

Hemodialysis patients (HDPs) exhibit extensive cardiovascular risk. The widely prescribed anti-platelet agent clopidogrel is metabolically activated by cytochrome enzymes, which may be impaired by uremia and chronic low-grade inflammation, typically present in HDPs. We conducted a prospective multicenter study to investigate the pharmacokinetics and pharmacodynamics of clopidogrel in HDPs and healthy volunteers (HVs).

**Methods:**

We enrolled HDPs receiving long-term clopidogrel (75 mg) and pantoprazole treatment (40 mg). Healthy volunteers received a loading dose of 300 mg clopidogrel, followed by 75 mg once daily. Pantoprazole, a substrate and probe drug of *CYP2C19*, was administered intravenously (40 mg). Plasma concentrations were quantified by mass spectrometry. Pharmacokinetics were calculated, and a population pharmacokinetic model was developed. The primary endpoint was the maximum concentration of clopidogrel’s active metabolite. Platelet aggregation was measured using adenosine diphosphate-induced whole-blood aggregometry.

**Results:**

Seventeen HDPs and 16 HVs were included. The maximum concentration of clopidogrel’s active metabolite was significantly lower in HDPs compared to HVs (median [interquartile range] 12.2 [4.6–23.4] vs. 24.7 [17.8–36.5] ng/ml, *P =* 0.02). The maximum concentration ratio of clopidogrel’s active metabolite to prodrug was 8.5-fold lower in HDPs, and an 82.7% reduced clopidogrel clearance, including clopidogrel’s active metabolite formation, was found using population pharmacokinetic modeling. From previous studies, adenosine diphosphate-induced platelet aggregation at 120 minutes was significantly higher in HDPs than in HVs (median [interquartile range]: 26 U [14 U–43 U] vs. 12 U [11 U–18 U], *P =* 0.004. Pantoprazole terminal half-life was ∼1.7-fold higher in HDPs compared to HVs.

**Conclusion:**

Our data demonstrate an altered metabolism of clopidogrel in HDPs in the context of lower *CYP2C19* activity, with potential implications for other substances metabolized by this enzyme.

## Graphical abstract

**Figure:**
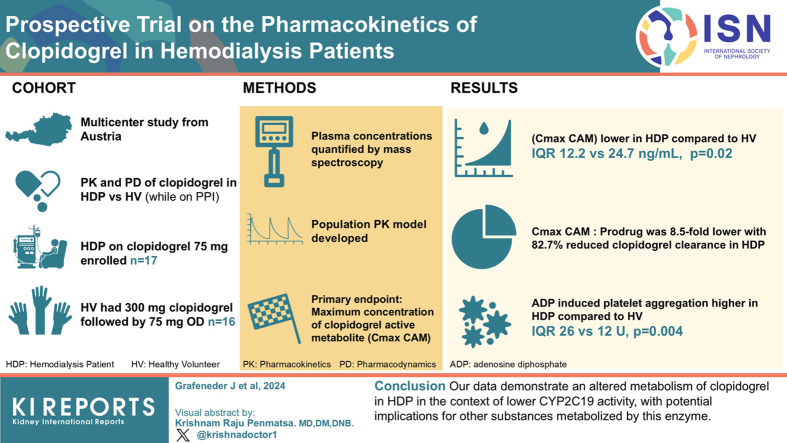

Hemodialysis patients (HDPs) exhibit substantial excess mortality with varying annual mortality rates depending on geographic, cultural, and socioeconomic differences.^1^ Cardiovascular disease is the primary cause of death in HDPs, accounting for more than 50% of deaths in this patient population.^2^^,^^3^

Antiplatelet drugs are important in secondary prophylaxis of cardiovascular disease in HDPs. In a systematic review, Natale *et al.*^4^ reported that antiplatelet agents reduced the risk of myocardial infarction at the cost of an increased risk of major bleeding. In HDPs, all-cause mortality and cardiovascular mortality were not reduced by the use of antiplatelet drugs, concluding that their clinical value in this specific cohort remains uncertain.

Rubin *et al.* reported that approximately two-thirds of all HDPs with clopidogrel have a high “on-treatment” platelet reactivity (HTPR).^5^ Interestingly, the authors found an increased rate of major adverse cardiovascular events in HDPs compared to non-HDPs (23.4% vs. 10.7%). The difference was more pronounced in HDPs with HTPR (28% vs. 16.5% in HDPs without HTPR). Mechanisms that may explain this high rate of HTPR include an increased rate of platelet turnover and altered pharmacokinetics (PK), more specifically, an impaired ability to activate the prodrug clopidogrel by *CYP2C19*.^6^, ^7^, ^8^ Ladda *et al.* reviewed cytochrome enzyme-mediated drug metabolism in HDPs and discussed uremic toxins or inflammatory cytokines as 2 possible mechanisms for the reduced activity of these enzymes.^9^

Despite reported HTPR to clopidogrel, evidence on the PK of clopidogrel or *CYP2C19* drug metabolism in HDPs is lacking.^7^ In acute inflammatory conditions, *CYP2C19* activity may be down-regulated, potentially causing reduced generation of clopidogrel’s active metabolite (CAM).^10^^,^^11^

Pantoprazole, a proton-pump inhibitor widely used for stress ulcer prophylaxis and gastroesophageal reflux disease,^12^ is metabolized mainly by *CYP2C19* and to a lesser extent by *CYP3A4*. Genetic variants in the *CYP2C19* enzymes substantially influence the PK of pantoprazole.^13^ In critically ill patients, the terminal elimination half-life was found to be roughly 5-fold increased, whereas there was a 2-fold increase in patients after cardiopulmonary resuscitation at the intensive care unit.^11^

We hypothesized that a combination of latent chronic inflammation, as frequently observed in patients with terminal kidney failure and hemodialysis, and uremia might be associated with reduced cytochrome P450 (CYP) metabolism in HDPs, leading to decreased exposure to CAM.^14^^,^^15^ We also sought to investigate the PK of pantoprazole as a second, intravenously available probe drug for *CYP2C19* activity in this population.

## Methods

This study received approval from the independent Ethics Committee at the Medical University of Vienna and the competent authorities (EC Nr: 2307/2019). It was registered within the European Union’s trial database (EudraCT 2019-004577-86). The study complied with the principles outlined in the Good Clinical Practice guideline and the Declaration of Helsinki.

### Study Population

We recruited 2 groups of participants for our study, i.e., healthy volunteers (HVs) and patients undergoing hemodialysis (HDP) who were at least 18 years old. HVs were enrolled at the Department of Clinical Pharmacology at the Medical University of Vienna. At the same time, HDPs were recruited at the Department of Nephrology and Dialysis at the Medical University of Vienna and the Department of Medicine VI for Nephrology and Dialysis at the Clinic Ottakring.

The inclusion criteria for HDPs were undergoing maintenance hemodialysis and receiving chronic treatment (≥1 week) with clopidogrel 75 mg per day. For HVs, the key inclusion criterion was normal renal function, as judged by the investigator. Participants with increased bleeding risk (platelet count < 100,000/μl) or hemoglobin <8 g/dl were excluded. A detailed list of the exclusion criteria for all participants can be found in the Supplementary Material. Before any trial-related activity was performed, we obtained written and oral informed consent from all participants.

### Study Design

HVs received a loading dose of 300 mg of clopidogrel on day 1, followed by 75 mg of clopidogrel once daily for 6 days, self-administered at home. On the morning of day 7, HVs reported to the study ward after an overnight fast. The last dose was administered at the study ward. Blood samples were collected at various time points, including baseline (before the last dose) and 15 minutes, 30 minutes, followed by 1, 2, 3, and 4 hours after intake of 75 mg clopidogrel and bolus infusion of 40 mg pantoprazole. HDPs continued their usual medication regimen, except for clopidogrel (75 mg), which they took under observation by the investigator after an overnight fast, and pantoprazole, which the investigator infused. Blood samples were obtained at the time mentioned above points directly from the arterial line of the dialysis machine (i.e., the line transporting venous blood from the patient to the hemodialysis machine). Plasma samples were collected after centrifugation at 2,000 g for 10 minutes at 4 °C. Aliquots of 500 μl were stored at –80 °C.

### Bioanalysis, Genetic Polymorphisms, and Biomarkers

Liquid chromatography-tandem mass spectrometry was used to measure plasma concentrations of clopidogrel and pantoprazole.^16^^,^^17^ The lower limit of quantification was 0.1 ng/ml for clopidogrel and CAM, and 0.05 mg/l for pantoprazole, respectively. *CYP2C19* polymorphisms (*CYP2C19*∗2, ∗3, or ∗17) were analyzed for all subjects as described previously.^18^ The *CYP2C19* polymorphism classification can be found in the Supplementary Material. C-reactive protein, interleukin-6, and serum amyloid A were analyzed as markers of chronic inflammation and to prevent the inclusion of participants with acute infections.

### Platelet Function Testing

Multiple Electrode Aggregometry on a Multiplate Analyzer (Dynabyte Medical) was used to determine whole blood aggregation. A detailed description of adenosine diphosphate-induced (ADP-induced) platelet aggregation can be found in previous studies.^11^^,^^17^ Adenosine diphosphate-induced platelet aggregation is expressed in Units [U], and platelet reactivity units (Platelet Reactivity Index [PRI]) are expressed in percentage [%]. Whole blood aggregometry was measured at predefined time points (baseline, 2, and 4 h). We defined HTPR using the recommended cut-off of >46 U (arbitrary units).^19^^,^^20^ Thrombin-receptor activating peptide-6 induced platelet aggregation was conducted to investigate overall platelet function and to exclude other forms of platelet disorders.^21^ The vasodilator-stimulated phosphoprotein phosphorylation assay specifically quantified the activation of the P2Y12 receptor. To measure the vasodilator-stimulated phosphoprotein phosphorylation assay, we used an enzyme-linked immune assay as described in previous studies.^11^^,^^22^ Based on the sensitivity to predict ischemic events^23^ and stent thrombosis,^20^ a PRI of >50% was chosen as the cut-off for HTPR.

### Pharmacokinetic and Statistical Analyses

The primary endpoint of this study was the maximum concentration of CAM (C_max_CAM_). Secondary endpoints included the HTPR rate in HDPs and HVs. Furthermore, the PK parameters of clopidogrel and pantoprazole were compared between HDPs and HVs. T-test, Mann-Whitney-*U*-test, and χ2-test were used for between-group comparisons, as applicable. A 2-tailed *P* value <0.05 was considered significant.

For graphical and statistical analysis, IBM SPSS version 23.0 (IBM corporation, Armonk, NY, USA), R version 4.0.0 (R Core Team. R Foundation for Statistical Computing, Vienna, Austria. https://www.R-project.org).) and GraphPad Prism version 9.4.0 (GraphPad Software Inc., San Diego, California, USA) were used.

#### Sample Size

The sample size calculation was based on C_max_CAM_ as the primary outcome parameter. Previous studies show a large range for C_max_CAM_ in healthy subjects.^24^, ^25^, ^26^ Given these results, we followed a rather conservative approach based on a study by Angiolillo *et al.*^26^ The mean value of C_max_CAM_ across all groups was 16.0 (SD 7.93). We expected C_max_CAM_ to be 8.04 in the dialysis group.^27^ Given these data, we calculated a minimum sample size of 32 (16 subjects per group) to provide 80% power to detect a 50% lower C_max_CAM_ in the HDP group compared to the healthy subjects with a 2-sided type I error rate of 0.05.

#### Pharmacokinetic Analyses

Non-compartmental analysis was performed using Phoenix WinNonlin (Certara, New Jersey, USA) to calculate pharmacokinetic parameters for clopidogrel and pantoprazole (C_max_, AUC [area under the curve], half-life, etc.). Additionally, the CAM to the prodrug ratio was determined for the Cmax and the AUC.

A population pharmacokinetic model was developed to describe the PK of clopidogrel and CAM jointly. Modeling activities are detailed in the Supplementary Material. In brief, 1- and 2-compartment models were evaluated for both clopidogrel and CAM, with first-order absorption of clopidogrel (with or without absorption delay). A liver compartment was included in the model to account for the first-pass metabolism of clopidogrel.^28^^,^^29^ To identify factors potentially causing differences in the pharmacokinetic parameters between patients, the study group (HV and HDP) and genotype were investigated as covariates on total clopidogrel clearance and body weight on all volume and clearance parameters.

## Results

### Study Population

A total of 16 HVs and 17 HDPs were recruited between June 2020 and October 2021. One HV was excluded due to a screening failure. Adverse events are summarized in the Supplement. Inflammation parameters were significantly higher in the HDP group (HDP/HV, median [interquartile range or IQR]: C–reactive protein: 0.66 [0.17–0.91]/0.09 [0.05–0.2] mg/dl, *P =* 0.003; –interleukin–6: 13.6 [10.5–21.3]/1.8 [1.6–2.7] pg/ml, *P <* 0.001; serum amyloid A: 13.1 [7.6–44.2]/4.2 [4.2–5.6] mg/L, *P <* 0.001, see Table 1 for all patient characteristics).

**Table 1:** Demographics and baseline characteristics

Parameter	HV *N* = 16	HDP *N* = 17
Age [yr]	56 (53–57)	65 (60–68)
Gender, male : female	12:4	14:3
Body mass index [kg/m^2^]	26.9 (25.4–30.9)	29.2 (22.4–30.5)
Time on hemodialysis [yr]	n.a.	2 (1–4)
Remaining diuresis [ml/d]	n.a.	0 (0–1200)
Etiology of ESRD, n (%)	n.a.	
Diabetic nephropathy		6 (35)
Vascular nephropathy		3 (18)
Glomerulonephritis		2 (12)
ADPKD		1 (6)
Other causes		5 (29)
Prior kidney transplantation, n (%)	n.a.	6 (35)
Clopidogrel monotherapy, n (%)	n.a.	6 (35)
Indication for Clopidogrel, n (%)		
Coronary artery disease		11 (65)
Cerebrovascular disease		0 (0)
Peripheral artery disease		7 (41)
Dual anti–platelet therapy, n (%)	n.a.	11 (65)
Concomitant therapy with Vitamin K antagonist, n (%)	n.a.	0 (0)
Platelets [G/l]	230 (210–243)	171 (135–239)
Leucocytes [G/l]	6.1 (4.64–6.79)	6.12 (4.98–6.62)
C–reactive protein [mg/dl]	0.09 (0.05–0.22)	0.66 (0.17–0.91)
Interleukin 6 [pg/ml]	1.8 (1.6–2.7)	13.6 (10.5–21.3)
Serum Amyloid A [mg/l]	4.2 (4.2–5.6)	13.1 (7.6–44.2)

ADPKD, Autosomal Dominant Polycystic Kidney Disease; AUC_last_, area under the curve until the last observed value; BMI, body mass index; ESRD, end–stage renal disease; f, female; HDPs, hemodialysis patients; HV, healthy volunteers; m, male; n.a., not applicable.

Blood values were taken before Clopidogrel was administered. Medians and (quartiles) are presented if not stated otherwise.

### Pharmacokinetic Analyses

**Figure 1:**
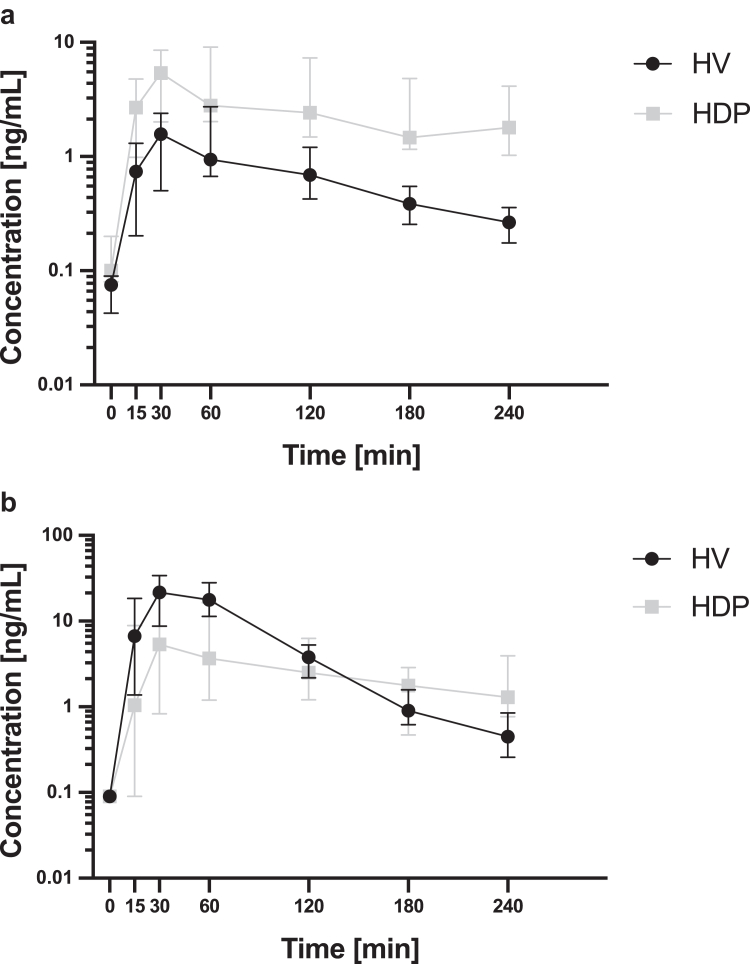
Concentration of clopidogrel as prodrug (a) and its active metabolite (b) over time. Data are presented as the median and the interquartile range. HDP, hemodialysis patients; HV, healthy volunteers.

**Figure 2:**
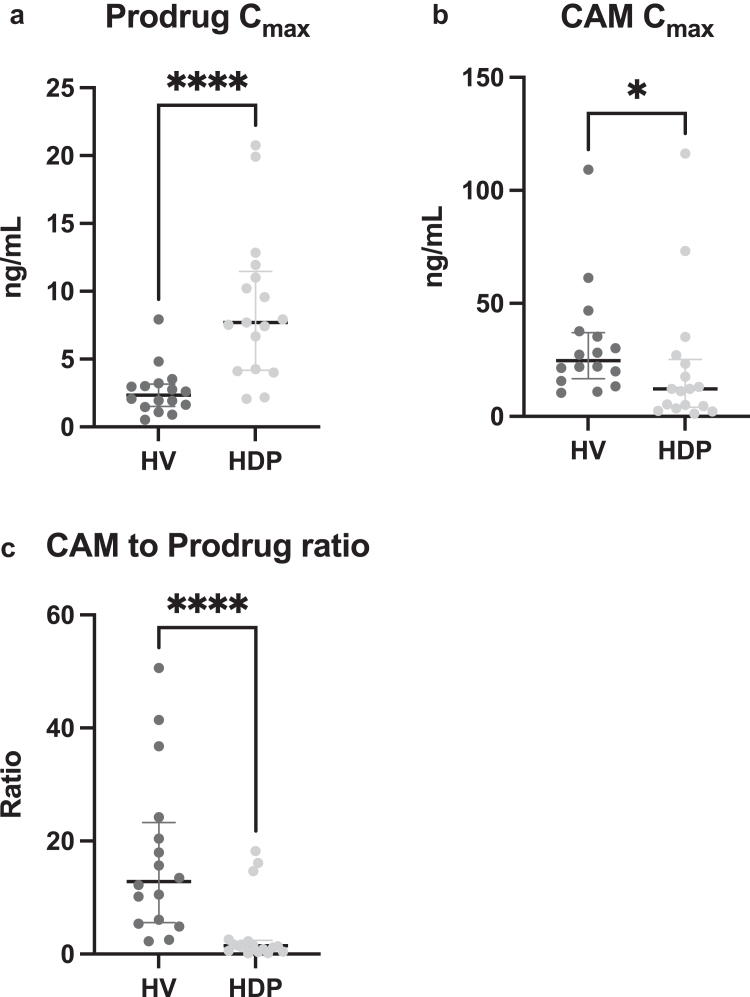
Concentration of the clopidogrel prodrug (a) and the active metabolite (CAM; b). (c) The ratio between CAM and prodrug can be seen. Horizontal lines represent the median and the interquartile range. CAM, active metabolite of clopidogrel; Cmax, maximum concentration; HDP, hemodialysis patients; HV, healthy volunteers; IQR, interquartile range;

Plasma concentration-time profiles (Figure 1) of clopidogrel and CAM revealed C_max_CLO_ to be 3.3-fold higher in the HDPs group compared to HVs (median [IQR]: 7.7 [4.3–11.0] vs. 2.3 [1.6–3.1] ng/ml, *P <* 0.001; see Table 2, Figure 2a). In contrast, C_max_CAM_ was 51% lower in the HDP group (median [IQR] 12.2 [4.6–23.4] vs. 24.7 [17.8–36.5] ng/ml in HV, *P =* 0.02; Figure 2b). In HDPs, the median C_max_ ratio CAM/prodrug was 1.5 (IQR 0.6–2.3), thus 8.5–fold lower compared to healthy subjects (median C_max_ ratio [IQR] 12.8 [5.7–22.3], *P <* 0.001, Figure 2c).

**Table 2:** Pharmacokinetics of clopidogrel and pantoprazole and results of genotyping

Parameter	HV *n* = 16	HDP *n* = 17
*Clopidogrel*		
C_max,CLO_ [ng/ml]	2.3 (1.6–3.1)	7.7 (4.3–11.0)
C_max,CAM_ [ng/ml]	24.7 (17.8–36.5)	12.2 (4.6–23.4)
C_max_ ratio CAM:CLO	12.8 (5.7–22.3)	1.5 (0.6–2.3)
AUC_last_CLO_ [ng/ml·min]	188 (140–279)	629 (525–1313)
AUC_inf_CLO_ [ng/ml·min]	227 (182–312)	810 (589–1192)
AUC_last_CAM_ [ng/ml·min]	1596 (1124–1897)	1057 (467–2021)
AUC_inf_CLO_ [ng/ml·min]	1760 (1408–2515)	1160 (477–9028)
t_1/2_CLO_ [min]	87.5 (63.7–131)	127 (90.0–149)
t_1/2_CAM_ [min]	35.2 (30.6–48.0)	74.1 (44.9–287)
*Pantoprazole*		
C_max_ [μg/ml]	9.6 (4.3–15.9)	2.9 (2.3–3.9)
AUC_last_ [μg/ml·min]	409 (316–472)	356 (266–391)
AUC_inf_ [μg/ml·min]	468 (333–527)	513 (370–674)
t_1/2_ [min]	76.8 (59.9–91.0)	130 (106–190)
Clearance [ml/min]	85.7 (69.0–122)	83.9 (60.1–249)
V_D_ [ml]	7528 (5025–11,705)	17,849 (14,665–33,658)
*Genotype, n (%)*		
Poor metabolizer	–	–
Intermediate metabolizer	7 (44)	3 (18)
Normal metabolizer	8 (50)	6 (35)
Rapid metabolizer	1 (6)	7 (41)
Ultrarapid metabolizer	–	1 (6)

AUC_inf_CLO_, AUC to infinity for clopidogrels prodrug; AUC_last,_ area under the curve until the last observed value; AUC_last_CAM_, AUC to the last observed value of colopidogrels active metabolite (CAM); BMI, body mass index; HDP, hemodialysis patients; HV, healthy volunteers; t_1/2_, half-life; t_1/2_CAM_, half-life of CAM; t_1/2_CLO_, half-life of clopidogrels prodrug; V_D_, volume of distribution.

AUC_last_CLO_ (area under the curve of the prodrug until the last observed value) was 3.3-fold higher in HDPs (median [IQR]: 629 [525–1313] vs. 188 [140–279] ng/ml·min in HVs, *P <* 0.001). However, AUC_last_CAM_ did not significantly differ between the 2 groups (HDP/HV, median [IQR]: 1057 [467–2021]/1596 [1124–1897] ng/ml·min, *P =* 0.292). There was no significant difference in the terminal elimination half–life of the prodrug between the groups (median [IQR] HDPs: 127 [90–149] vs. 88 [64–131] min in HVs, *P =* 0.357), whereas this parameter significantly differed for CAM (HDP/HV, median [IQR]: 74 [45–287]/35–30, 31, 32, 33, 34, 35, 36, 37, 38, 39, 40, 41, 42, 43, 44, 45, 46, 47 min, *P =* 0.003; Table 3).

**Table 3:** Pharmacokinetics of clopidogrel, its active metabolite and pantoprazole, grouped by genotype

	HV			HDP			
	IM	NM	RM	IM	NM	RM	UM
*n*	7	8	1	3	6	7	1
*Clopidogrel*							
C_max_CLO_ [ng/ml]	1.9 (1.1–2.1)	3.1 (2.9–4.2)	1.5	4 (2.1–9.6)	7.6 (4.1–7.9)	10.2 (6.7–19.9)	11
C_max_CAM_ [ng/ml]	22.1 (13.3–30.1)	24.8 (17.8–49.5)	35.4	5.3 (1.2–73.2)	12.2 (3.6–35.2)	12.2 (5.1–23.4)	4.6
C_max_ ratio CAM:CLO	13.5 (10.5–18)	8.1 (3.7–28.6)	24.2	2.6 (0.1–18.2)	1.6 (0.9–14.7)	1.4 (0.6–1.6)	0.4
AUC_last_CLO_ [ng/ml·min]	160 (94–195)	279 (193–461)	127	389 (184–1599)	628 (525–1068)	641 (594–1729)	1686
AUC_last_CAM_ [ng/ml·min]	1601 (987–1929)	1354 (1124–2452)	1706	994 (527–1731)	156 (1044–2341)	1057 (458–4794)	129
t_1/2_CLO_ [min]	87.5 (66.1–123)	82 (60.7–139)	131	132 (132–132)	81.8 (65.6–121)	138 (112–150)	∗
t_1/2_CAM_ [min]	33 (30.6–42.4)	43.2 (35–53.2)	24	161 (35.8–287)	49.2 (42.6–149)	197 (59.5–384)	∗
Pantoprazole							
C_max_ [μg/ml]	12 (3.6–16.7)	6.8 (4.3–13.3)	18.7	3.9 (0.8–4.3)	3.2 (2.3–4.1)	2.7 (1–3.1)	3.4
AUC_last_ [μg/ml·min]	423 (359–537)	363 (265–442)	492	391 (41.3–500)	417 (281 – 500)	329 (56–362)	306
t_1/2_ [min]	83.9 (64.9–97.5)	69.3 (55.8–92.3)	57.3	129 (39.2–285)	195 (131–219)	112 (51.4–134)	124
Clearance [ml/min]	81.9 (59–99.1)	99.8 (65–144)	79	61.2 (58.1–100)	89.7 (77.9–91.9)	78 (27.4–389)	790
V_D_ [ml]	8484 (5923–12,003)	7682 (3987–11,705)	4128	17,774 (16,415–17,923	15,619 (11,837–21,927	20,376 (14,061–52,852)	40,609
*Multiplate*							
ADP [U]	15 (12–18)	11 (8–20)	6	34 (15–87)	14 (13–28)	27 (17–44)	21
VASP PRI							
0 min [%]	71 (59–80)	41 (25–53)	15	60 (36–71)	44 (29–66)	70 (57–73)	75
	69 (46–76)	36 (32–46)	23	82 (73–85)	53 (19–78)	72 (70–77)	75
	70 (47–78)	40 (30–65)	24	90 (77–92)	39 (15–49)	68 (56–74)	84

ADP, adenosine diphosphate; CAM, clopidogrel’s active metabolite; C_max_, maximum concentration; HDP, hemodialysis patients; HV, healthy volunteers; IM, intermediate metabolizer; NM, normal metabolizer; PRI, platelet reactivity index; RM, rapid metabolizer; UM, ultrarapid metabolizer; VASP, vasodilator–associated stimulated phosphoprotein; ∗, could not be calculated.

Data is presented as medians and interquartile ranges.

The final population PK model included first-order clopidogrel absorption with lag time, 2 compartments to describe clopidogrel PK and 1 compartment for CAM. Total clopidogrel clearance, including CAM formation, was 82.7% lower in HDPs than in HVs. Estimating separate CL_CLO_ parameters for the different genotype groups (rather than patient groups) was not supported by the model. Furthermore, no significant influence of the study groups on the central volume of distribution (V_D_) of clopidogrel or fm, that is, the fraction of the clopidogrel dose converted to CAM, was found. Detailed results, including model parameter estimates, are presented in the Supplementary Material (Table S1, Supplementary Figures S1–S4).

The terminal elimination half-life of pantoprazole was significantly higher in HDPs (median [IQR]: 130 [106–190] vs. 77 [60–91] min in HVs, *P =* 0.019, Supplementary Figure S7). The V_D_ was markedly higher in HDPs (median [IQR]: 17,849 [14,665–33,658] vs. 7528 [5025–11,705] ml in HVs, *P <* 0.001).

### Platelet Function Testing

**Figure 3:**
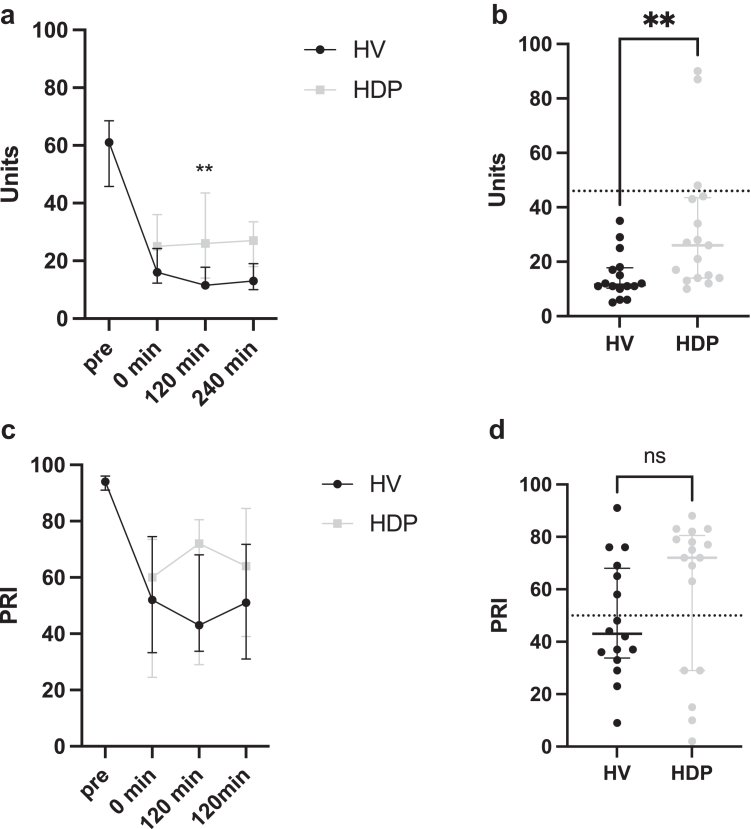
Results for Multiplate assay (ADP-induced aggregation) and VASP assay (Platelet-reactivity index, PRI) over time (a, c). Individual data are shown at 120 min (b, d). Data are presented as median and interquartile range. The dotted lines in b and d represent the HTPR cutoff (>46 U for ADP and > 50% for VASP). ∗∗*P <* 0.01; ADP, adenosine diphosphate; HDP, hemodialysis patients; HTPR, high on-treatment platelet reactivity; HV, healthy volunteers; PRI, platelet reactivity index; VASP, vasodilator-associated stimulated phosphoprotein;

According to whole blood aggregometry (Figure 3), 3 individuals in the HDPs cohort (18%) displayed HTPR, whereas none of the HVs had HTPR 120 min after clopidogrel intake (*P =* 0.078). Adenosine diphosphate-induced platelet aggregation was significantly higher in the HDPs at 120 min (HDP/HV, median [IQR]: 26^14^, ^15^, ^16^, ^17^, ^18^, ^19^, ^20^, ^21^, ^22^, ^23^, ^24^, ^25^, ^26^, ^27^, ^28^, ^29^, ^30^, ^31^, ^32^, ^33^, ^34^, ^35^, ^36^, ^37^, ^38^, ^39^, ^40^, ^41^, ^42^, ^48^/12^11^, ^12^, ^13^, ^14^, ^15^, ^16^, ^17^, ^18^; *P =* 0.004, Figure 3b). Thrombin-receptor activating peptide-6-induced platelet aggregation showed no significant difference between groups (HDP/HV, median [IQR]: 110 [99–119]/70 [64–100] U).

The PRI (HDP/HV, median [IQR]: 72 [29%–79%] /43% [35%–67%]; *P =* 0.17) did not differ significantly between groups at 120 min after clopidogrel intake (Figure 3). Likewise, the HTPR rate according to the PRI (HDPs (*n* = 12)/HV (*n* = 6) [95% CI]: 71% [47%–87%] /38% [18%–61%]; *P =* 0.056) did not differ significantly between groups, albeit a trend towards a higher PRI in the HDPs group could be noted. Associations of the genotype with platelet inhibition were most prominent for PRI in HVs (Supplementary Figures S5 and S6).

## Discussion

We investigated the PK and pharmacodynamics of clopidogrel and pantoprazole in HDPs and HVs. Our findings showed significantly altered PK of both drugs in HDPs.

The C_max_ CAM to prodrug ratio was 8.5 times higher in HVs than in HDPs, presumably due to faster metabolization. Population PK modeling revealed an 82.7% lower total clearance, including CAM formation rate, in HDPs compared to HVs. In contrast, and surprisingly, at first sight, no significant difference was found between the genotypes. However, genotype patterns were not equally distributed in the 2 study groups, with intermediate metabolizers (IM) mainly present in HVs and rapid and ultrarapid metabolizers almost exclusively found in the population of HDPs (rapid metabolizer/ultrarapid metabolizer: 88.9%, normal metabolizer: 42.9%, IM: 30% HDPs). Given this imbalance, the 82.7% clearance reduction estimate can be considered conservative.

For pantoprazole, HDPs displayed a significantly higher V_D_ than HVs did, whereas the AUC and the clearance were comparable between both groups. Of note, 85% of the predose samples taken for pantoprazole were below the limit of quantification, limiting the ability to accurately describe pantoprazole clearance, particularly given the short observation window. We found a ∼1.7-fold longer terminal elimination half-life in HDPs compared to HVs, probably driven mainly by the markedly higher V_D_ in HDPs. Of note, Kliem *et al.* ^48^ also reported an increased V_D_ in HDPs similar to our results. Increased V_D_ in kidney disease has previously also been reported for other drugs.^30^ We also found increased pantoprazole V_D_ in critically ill patients after resuscitation.^31^ The median half-life of pantoprazole in HVs was 1.3 hours, comparable to other studies.^32^ The half-life of pantoprazole in HDPs was ∼1.7 fold longer (2.2 hours). Kliem *et al.* ^48^ compared the PK of pantoprazole in 8 patients undergoing regular hemodialysis on days with and without hemodialysis and found no significant differences. However, compared with healthy subjects, they even found a shorter half-life. The authors attributed this effect to a lower protein binding than in healthy subjects (96% vs. 98%), possibly caused by the use of unfractionated heparin. However, there is no data about his hypothesized interaction between unfractionated heparin and pantoprazole. Patients in our study routinely received low-molecular-weight heparin, and we did not quantify protein binding. In addition, our study’s terminal elimination half-life of pantoprazole is comparable with other studies.^13^

Inflammation, which commonly occurs in HDPs, had previously been investigated as a potentially influential factor for CYP expression in a PK study on simvastatin in rheumatoid arthritis. Simvastatin exposure significantly decreased 1 week after tocilizumab treatment, suggesting improved *CYP3A4* activity, most likely caused by the anti-inflammatory effects of tocilizumab.^33^ Although our study excluded participants with acute inflammatory conditions, the study population included patients with chronic low-grade inflammation, common in HDPs.^34^ Our findings might also apply to other diseases that feature chronic low-grade inflammation, such as rheumatoid arthritis, inflammatory bowel disease, or chronic obstructive pulmonary disease.^35^^,^^36^

In murine models of chronic renal failure, a reduced expression of CYP2C11, CYP3A1, and CYP3A2 was demonstrated^37^ Furthermore, *in vitro* data indicated direct inhibitory effects of some small molecular weight uremic toxins on CYP activity.^38^ Therefore, a contribution of uremic toxins to impaired CYP activity seems possible. In our study, we did not measure uremic toxins. It is important to note that *in vivo* inflammation and uremia are commonly coexistent in HDPs, and their effects are very difficult to differentiate.^15^^,^^39^

In critically ill patients with more pronounced inflammatory conditions (median C-reactive protein 12.8 mg/dl) and varying renal function, an even more pronounced impaired metabolism of clopidogrel and pantoprazole was shown (∼5-fold increase of pantoprazole half-life and ∼30-fold decreased CAM/prodrug ratio).^11^ Mostowik *et al.* ^40^ demonstrated that the platelet-inhibitory effects of clopidogrel were negatively associated with biomarkers of inflammation in patients with stable coronary artery disease who underwent a percutaneous coronary intervention but did not consider uremic toxins as a potentially influential factor on the response.

Only 3 HDPs (17.6%) but no HVs had HTPR according to whole blood aggregometry, the latter being in line with previous reports.^41^ After clopidogrel intake, ADP-induced aggregation was significantly higher in the HDPs group (Figure 3b), together with higher variability within the HDPs group. Interestingly, 12 (71%) HDPs and 6 (50%) HVs displayed HTPR according to the VASP assay. Overall, the P2Y12 receptor-specific VASP assay also yielded significantly less inhibition in HDPs than in HVs. The significant discrepancy between these 2 assays has been reported previously.^42^, ^43^, ^44^, ^45^, ^46^ We measured thrombin-receptor activating peptide-6-induced platelet aggregation to exclude overall defective platelet aggregation and found comparable results between the groups. Guo *et al.* ^47^ found that a P2Y12 receptor-specific assay (VerifyNow) increased with the degree of kidney failure, whereas it did not impact Multiplate aggregometry. Thus, the difference between the P2Y12-receptor-specific assay and the rather unspecific ADP-induced platelet aggregation assessed by Multiplate was large in HDPs. Uremic toxins may affect ADP-induced platelet aggregation and the VASP assay differently. However, there is still a lack of data on that matter.

While the extraordinarily high cardiovascular risk in HDPs is well-known, data on the importance of adequate platelet inhibition are inconsistent. A meta-analysis found that patients receiving ticagrelor or prasugrel had significantly fewer major adverse cardiovascular events than clopidogrel-treated patients.^49^ Similarly, Park *et al.* ^50^ reported that intensified antiplatelet therapies were superior to clopidogrel in terms of preventing major adverse cardiovascular events in patients with chronic kidney disease, including HDPs. In contrast, another meta-analysis comparing the efficacy and safety of clopidogrel and ticagrelor HDPs found no significant differences between the 2 treatments.^51^ In a substudy of the ADAPT-DES study, HDPs with high residual platelet reactivity despite clopidogrel intake were at significantly higher risk of experiencing a major adverse cardiovascular event than patients with adequate platelet inhibition.^5^ Our HDP cohort consisted of clinically stable patients, whereas those with acute conditions, including acute inflammatory responses, were not eligible for our study. It is possible that the relatively low rate of HTPR and the mostly adequate inhibition of platelet aggregation observed in our study may be subject to a certain selection bias. The PK of other platelet inhibitors, first and foremost ticagrelor, are more stable and not as dependent on CYP metabolism as clopidogrel. Hence, our results may be cautiously interpreted as being supportive of the clinical data that show possible advantages of other P2Y12 inhibitors over clopidogrel. However, we have not collected clinical outcome data or performed direct comparisons between different P2Y12 inhibitors.

Some limitations of the present study should be acknowledged. First, due to its limited sample size, the study was exploratory. Second, the distribution of CYP2C19 metabolizer categories was uneven in the 2 study groups, that is., HVs and HDPs. CAM concentrations were previously shown to depend on the genetically determined metabolic activity of *CYP2C19*. (Ultra-)Rapid and extensive metabolizers displayed higher AUC_CAM_ values than intermediate and poor metabolizers^52^ In our study, (ultra)rapid metabolizers strongly dominated in the HDPs (rapid metabolizer/ultrarapid metabolizer: 88.9%), whereas there were more normal and poor metabolizers in the HVs. This unbalanced distribution of genotypes may have resulted in an underestimated reduction of clopidogrel metabolism. Third, the lack of testing for P2Y12 receptor polymorphism presents a limitation. Furthermore, the blood sampling schedule was restricted to 4 hours, during which patients underwent routine hemodialysis. This short period enabled the capture of only the first part of the concentration-time profiles and may have impacted the determination of pharmacokinetic measures, especially of clearance and AUC. The half-life of clopidogrel and its active metabolite in our study is in line with many other studies^11^^,^^26^^,^^27^ but differs significantly from the terminal elimination half-life published in official documents of about 6 hours. However, this may be explainable by different observation periods. Our focus was not to quantify the terminal elimination half-life of clopidogrel but to analyze the generation of the active metabolite, which mostly takes place within a few hours. Next, although pantoprazole is considered the proton pump inhibitor with the lowest potential for drug-drug interactions, a *CYP2C19* interaction with clopidogrel could not be fully ruled out. However, all study participants received the same doses of the 2 drugs. Finally, the protein binding of pantoprazole was not quantified in the study, and nor were other inactive metabolites of clopidogrel.

In summary, our data demonstrate that the PK and pharmacodynamics of drugs with a high level of CYP interaction, such as clopidogrel and pantoprazole, may be significantly altered in patients with chronic kidney disease undergoing hemodialysis. Further clinical trials in this population are warranted, especially with regard to drugs that are extensively metabolized via CYP enzymes.

## Disclosure

All the authors declared no competing interests.

## Supplementary Material

Supplementary material (PDF) Table S1:xxx.Figure S1: xxx. Figure S2: xxx. Figure S3: xxx. Figure S4: xxx. Figure S5: xxx. Figure S6: xxx. Figure S7: xxx. Supplementary references. CONSORT statement

## Footnotes

Supplementary material (PDF)
Table S1: Parameter estimates of the final population pharmacokinetic model. Figure S1: Deviations (ETA) of individual parameters in the population from the respective typical mean parameter - Base Model. Figure S2: Deviations (ETA) of individual parameters in the population from the respective typical mean parameter - Final Model. Figure S3: Goodness-of-fit plots for the population pharmacokinetic model. Figure S4: Visual predictive checks. Figure S5: Maximum concentration of the clopidogrel active metabolite grouped by genotype. Figure S6: Platelet inhibition grouped by genotypes. Figure S7: The half-life, volume of distribution, and concentration of pantoprazole over time. Supplementary references CONSORT statement
