# Supplementary material for: Prospective Trial on the Pharmacokinetics of Clopidogrel in Hemodialysis Patients
Source: Kidney Int Rep. 2024 Jul 31;9(10):2970–80. doi: 10.1016/j.ekir.2024.07.029 (PMC11489422; doi:10.1016/j.ekir.2024.07.029)
Supplement: Supplementary material (PDF) — Table S1:xxx.Figure S1: xxx. Figure S2: xxx. Figure S3: xxx. Figure S4: xxx. Figure S5: xxx. Figure S6: xxx. Figure S7: xxx. Supplementary references. CONSORT statement [file mmc1.pdf]

Supplementary Material

Kidney International Reports

Prospective Trial on the Pharmacokinetics of Clopidogrel in Hemodialysis Patients

Juergen Grafeneder[1,2], Wisse van Os[1], Iris K. Minichmayr[1], Katarina D Kovacevic Miljevic [1], Birgit Reiter[3], Marcus D. Säemann[4], Veronika Machold-Fabrizii[4], Amro Ahmed[5], Paul Spechtl[6], Haris Omic[6], Raute Sunder-Plaßmann[3], Bernd Jilma[1], Christian Schoergenhofer[1*#], and Farsad Eskandary[6#]

[1] Department of Clinical Pharmacology, Medical University of Vienna, Austria
[2] Department of Emergency Medicine, Medical University of Vienna, Austria
[3] Department of Laboratory Medicine, Medical University of Vienna
[4] 6th Medical Department, Nephrology and Dialysis, Clinic Ottakring, Vienna, Austria
[5] 3rd Medical Department, Cardiology and Intensive Care Medicine, Clinic Ottakring, Vienna, Austria
[6] Department of Nephrology and Dialysis, Division of Medicine III, Medical University of Vienna, Austria

***Corresponding Author:**
Christian Schoergenhofer
Department of Clinical Pharmacology
Medical University of Vienna, Austria
christian.schoergenhofer@meduniwien.ac.at
Tel.: +43 1 40400 29800

[#]Contributed equally

Inclusion/Exclusion Criteria

Inclusion:

- General:
  - aged ≥ 18
  - male or non-pregnant female
  - understanding of the purpose of the study

- Hemodialysis patients (HDP):
  - patients ≥18 undergoing maintenance hemodialysis receiving chronic treatment (i.e. ≥ 1 week) with clopidogrel 75 mg once per day

- Healthy volunteers (HV):
  - normal renal function, as judged by the investigator
  - matched by age, and sex to subjects included in the MHD group

Exclusion:

- increased bleeding risk (platelet count < 100,000/µL) or hemoglobin < 8 g/dL,
- concomitant therapy with potent CYP2C19 inhibitors, inducers, or substrates with a narrow therapeutic index within 14 days of study initiation
- history of alcohol, substance, or drug abuse within the year preceding the study, and
- clinically significant laboratory abnormalities as judged by the investigator
- pregnancy
- lactation and
- acute infection or inflammation as deemed by the treating physicians/study personnel.

CYP2C19 polymorphisms classification

We classified *1/*1 carriers as normal (NM), *1/*2 and *2/*17 carriers as intermediate (IM), *2/*2 carriers as poor (PM), *1/*17 carriers as rapid (RM), and *17/*17 carriers as ultrarapid (UM) metabolizers. CYP2C19*3 was not detected in the study participants.

Adverse Events

Within the healthy control group, three participants experienced headaches; all were deemed unrelated to the study. These headaches resolved spontaneously in less than 24 hours, with only one individual opting for self-administered ibuprofen (400 mg). Additionally, there was one instance of a participant reporting two days of depressive mood, which resolved without any intervention.

Within the HDP group, one patient reported pain in the left leg. However, this adverse event had already been reported several times before the study without a pathologic finding, so it was determined to be unrelated to the study.

Population pharmacokinetic modelling

Methods

To consider the formation of CAM from clopidogrel in the model, clopidogrel and CAM doses and concentrations were converted to molar units based on their respective molecular weights prior to modeling. Clopidogrel absorption after oral administration was modeled as a first-order process and evaluated with and without a lag time to consider absorption delay. The bioavailability of clopidogrel was assumed to be 100%, and the estimated parameters should be interpreted as apparent oral PK parameters. One- and two-compartment models were explored to describe both clopidogrel and CAM pharmacokinetics.

To account for the first-pass metabolism of clopidogrel, a liver compartment was included in the model between the absorption and central compartment, as previously suggested [S1, S2]. The volume of the hepatic compartment was set to be the same as the central volume. In the hepatic compartment, clopidogrel could be transformed into CAM or eliminated via other metabolic processes. Clopidogrel conversion to metabolites was assumed to be complete, and the fraction of the total clopidogrel clearance resulting in the formation of CAM was described using the parameter *fm*. The population estimate of *fm* was fixed at 0.125 according to previous evidence due to reasons of identifiability [S1]. The individual *fm* values were constrained between 0 and 1 using a logit transformation.

Inter-individual variability was evaluated for all structural model parameters using an exponential function, assuming a log-normal distribution of the individual PK parameters. Residual unexplained variability (RUV) was considered using additive, proportional, and combined additive and proportional models. Separate models were used to capture the RUV for clopidogrel and CAM. For clopidogrel and CAM, 11.3% and 16.0% of the observed concentrations were below the lower limit of quantification (BLQ). As the M3 method resulted in convergence issues, the M1 method was taken to handle BLQ values; i.e., BLQ values were removed [S3].

Study group and genotype were investigated as categorical covariates potentially influencing clopidogrel pharmacokinetics by estimating additional parameters capturing fractional differences between these subgroups (e.g., $CL_{healthy}$ and $CL_{hemodialysis}$). When using genotype as a covariate, rapid (n=8 individuals) and ultra-rapid (n=1) metabolizers were grouped together. We assessed a potential impact of body weight on the distribution and clearance parameters using allometric scaling with fixed exponents of 0.75 and 1 for clearances and volumes, respectively.

Model evaluation and selection was guided by statistical significance (difference in objective function value ΔOFV=3.84, α=0.05, *df=1* for nested models), precision and plausibility of parameter estimates, as well as goodness-of-fit plots including observed versus population/individual predicted concentrations, individual predicted concentration-time profiles, and conditional weighted residuals versus population predictions and time, stratified by compound (parent drug and metabolite) and patient group (healthy and hemodialysis). The predictive performance of the model was assessed by visual predictive checks stratified by compound (n=1000 simulations).

Population pharmacokinetic modelling was performed using NONMEM7.4 (ICON Clinical Research LLC, Gaithersburg, MD) and the first-order conditional estimation method (with interaction), assisted by PsN 5.3.9 (Uppsala University, Sweden; https://uupharmacometrics.github.io/PsN) and Pirana 21.11.1 (Certara USA, Inc). Graphical analyses were conducted using R4.2.2 (R Core Team. R Foundation for Statistical Computing, Vienna, Austria. https://www.R-project.org).

Results

Clopidogrel absorption was described by a first-order process with lag time. A two-compartment model best captured the pharmacokinetics of clopidogrel. For CAM, a one-compartment model was chosen, as an additional second compartment resulted in convergence issues of the model.

A significant difference in clopidogrel elimination was found between the two study groups (ΔOFV=27.1), with HDP showing 82.7% lower total clearance, and thus CAM formation rate, compared to healthy volunteers. Consideration of this difference in the model markedly decreased ('explained') interindividual variability (IIV) associated with CL_{CLO} [IIV_CL_{CLO} 86 versus coefficient of variation (CV) of 166%]. Furthermore, for a model allowing distinct CL_{CLO} for HV and HDP, no obvious difference was visible in plots depicting individual (empirical Bayes) estimates of CL_{CLO} for the different genotype groups (Figure S2). Before inclusion of the CL_{CLO}-patient group covariate relationship in the model, the same plots had surprisingly revealed highest median CL_{CLO} estimates for intermediate metabolizers (Figure S1), probably as this group included most HV (70%), while rapid and ultra-rapid metabolizers included 89% HDP. Estimating separate CL_{CLO} parameters for the different genotype groups was not supported with respect to statistical significance and diagnostic plots. Allometric scaling did not result in a considerable improvement and was thus not included in the final model.

Three observations (n=2 for clopidogrel; n=1 for CAM) in the dataset appeared implausible. Although supposed to have been measured just before administration of the study drug (i.e., at the end of a 24-h dosing interval), these concentrations were high relative to trough concentrations of other patients and, importantly, considerably higher relative to concentrations measured at 4 hours post-dose in the same patients. The concentrations were initially considered for modelling, though found to markedly distort the individual predictions for

the two individuals of concern. Furthermore, inclusion of the three concentrations for modelling inflated the parameters quantifying IIV, particularly variability in V_{C_CLO} (central volume of distribution of the prodrug; highest individual value was found in the HV cohort with two implausible concentrations: V_{C_CLO}=50,629 L compared with next highest individual value: V_{C_CLO}=10,739 L). The final model was thus based on a dataset excluding the three outlying concentrations. Removal considerably improved the individual fits for the two individuals in question, while the effect on the overall model performance (e.g. predictive performance) was moderate. The estimates of the model parameters are presented in Table S1, and the diagnostic plots and visual predictive checks are presented in Figure S3 and S4, respectively.

Table S1. Parameter estimates of the final population pharmacokinetic model

Parameter	Description	Estimate (RSE%)
**Clopidogrel**		
k_a (h^{-1})	Absorption rate constant	2.40 (13.5)
T_{lag} (h)	Absorption lag time	0.122 (11.0)
CL_{CLO} (L/h)	Clearance	
$CL_{CLO,HV}$ (L/h)	CL in healthy volunteers	18,100 (17.6)
$COV\_CL_{CLO,HD}$ [a]	Relative difference in CL for hemodialysis patients	-0.827 (5.3)
$V_{C,CLO} = V_{H,CLO}$ (L)	Central and hepatic volume of distribution	1,930 (19.7)
$V_{P,CLO}$ (L)	Peripheral volume of distribution	5,560 (13.2)
Q_C (L/h)	Intercompartmental CL between hepatic and central compartment	4370 (14.1)
Q_P (L/h)	Intercompartmental CL between central and peripheral compartment	3,090 (13.4)
IIV CL_{CLO} (CV%)	Interindividual variability in CL_{CLO}	86.3 (13.3)
IIV $V_{C,CLO}$ (CV%)	Interindividual variability in $V_{C,CLO}$	98.9 (15.2)
$RUV_{prop,CLO}$ (CV%)	Proportional residual unexplained variability	43.9 (7.2)
$RUV_{add,CLO}$ (nM)	Additive residual unexplained variability	0.306 (15.3)
**CAM**		
fm	Fraction of clopidogrel dose metabolized to CAM	0.125 FIX
CL_{CAM} (L/h)	Clearance	332 (12.8)
V_{CAM} (L)	Volume of distribution	89.9 (20.6)
IIV_fm (CV%)	Interindividual variability in fm	137.3 (13.4)
$RUV_{prop,CAM}$ (CV%)	Proportional residual unexplained variability	49.7 (7.0)

[a] $CL_{CLO} = CL_{CLO,HV} \cdot (1 + COV\_CL_{CLO,HD} \cdot HD)$ with HD = 1 for hemodialysis patients and HD = 0 for healthy volunteers. RSE: relative standard error; CV%: coefficient of variation, calculated as $\sqrt{e^{\omega^2} - 1} \cdot 100\%$; CAM: clopidogrel active metabolite

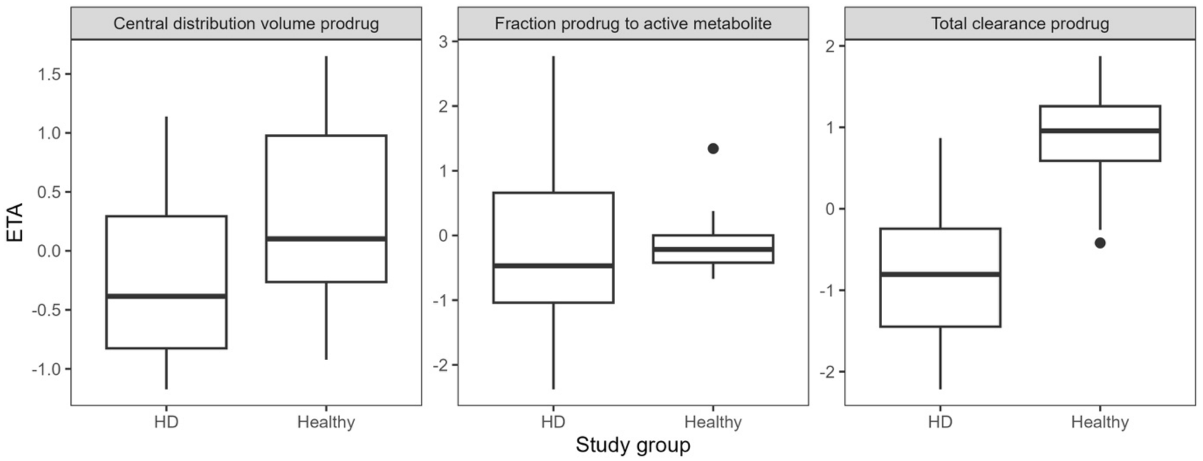

Figure S1. Deviations (ETA) of individual parameters in the population from the respective typical mean parameter, indicating interindividual variability, obtained from the *base* model (i.e., before inclusion of the covariate patient group on total clopidogrel clearance). HD: hemodialysis

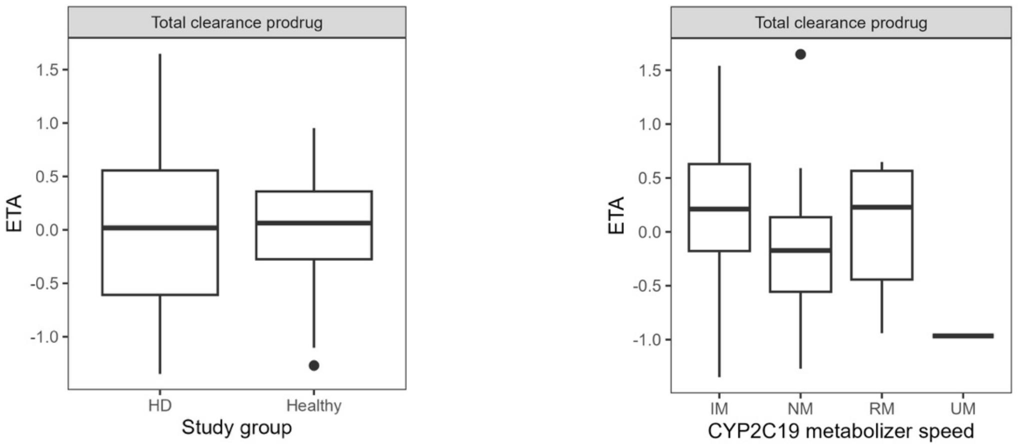

Figure S2. Deviations (ETA) of individual parameters in the population from the respective typical mean parameter, indicating interindividual variability, obtained from the *final* model (i.e., after inclusion of the covariate patient group on total clopidogrel clearance). HD: hemodialysis

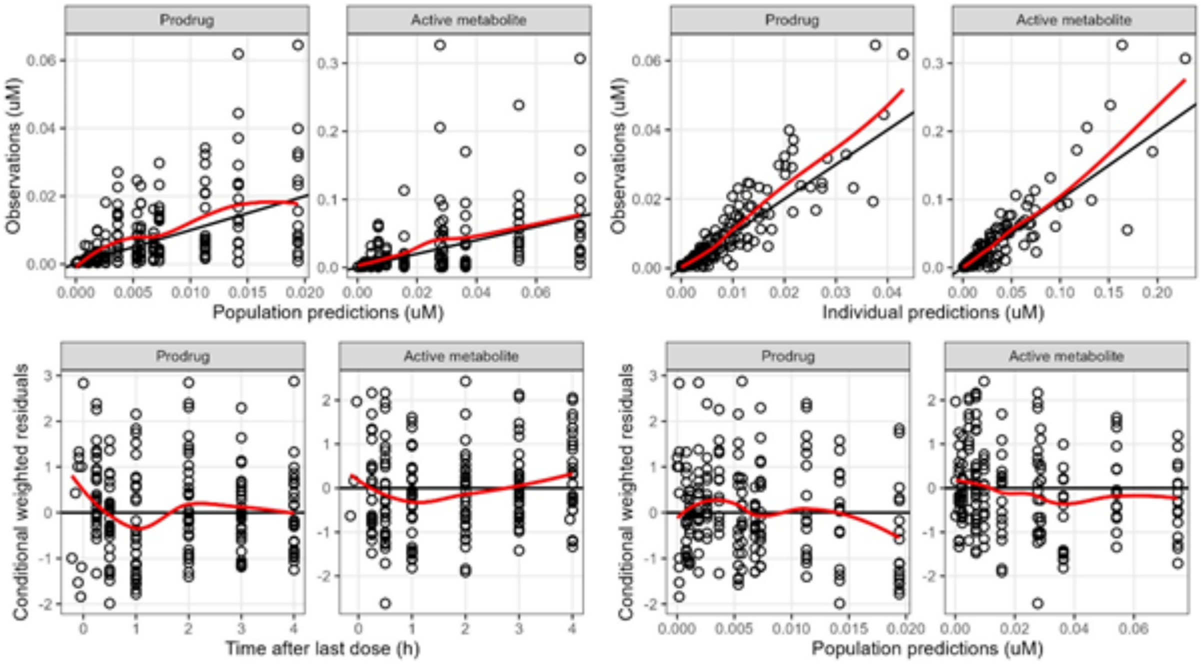

Figure S3. Goodness-of-fit plots for the population pharmacokinetic model: observed versus population predicted concentrations (upper left panels) and individual predicted concentrations (upper right panels) of the prodrug and active metabolite. Lower row: conditional weighted residuals (CWRES) versus time after last dose (lower left panels) and population predicted concentrations (lower right panels). Open circles represent observed concentrations. The solid black lines represent the line of unity (upper row) or a reference line (lower row); red solid lines depict smooth lines

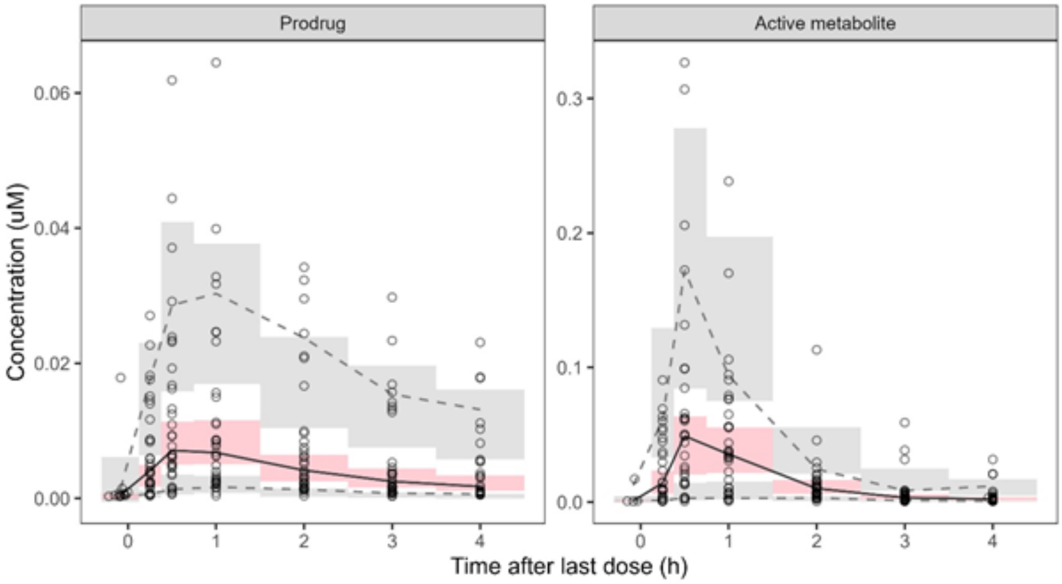

Figure S4. Visual predictive checks (n=1000) for the observed concentrations (open circles, median=solid black line) of clopidogrel (prodrug; left panel) and the active metabolite CAM (right panel). Dashed lines indicate the 10th and 90th percentile of the observed data. Shaded

areas show the 95% confidence intervals for the 10th, 50th, and 90th percentile of the simulated data

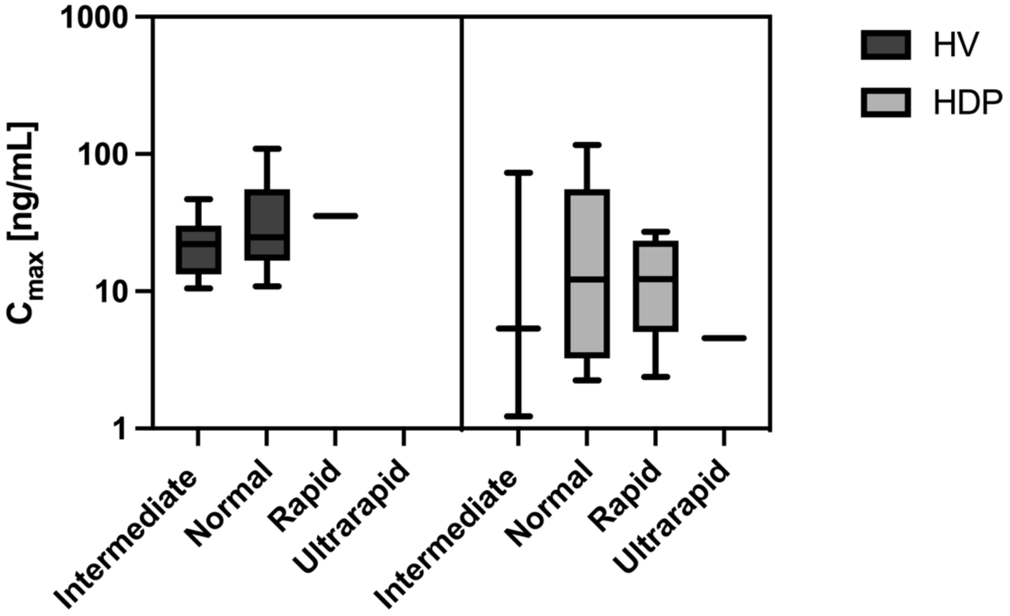

Figure S5. Maximum concentration (C_{max}) of the clopidogrel active metabolite (CAM) grouped by genotype (log scale). HV, healthy volunteers; HDP, hemodialysis patients**.**

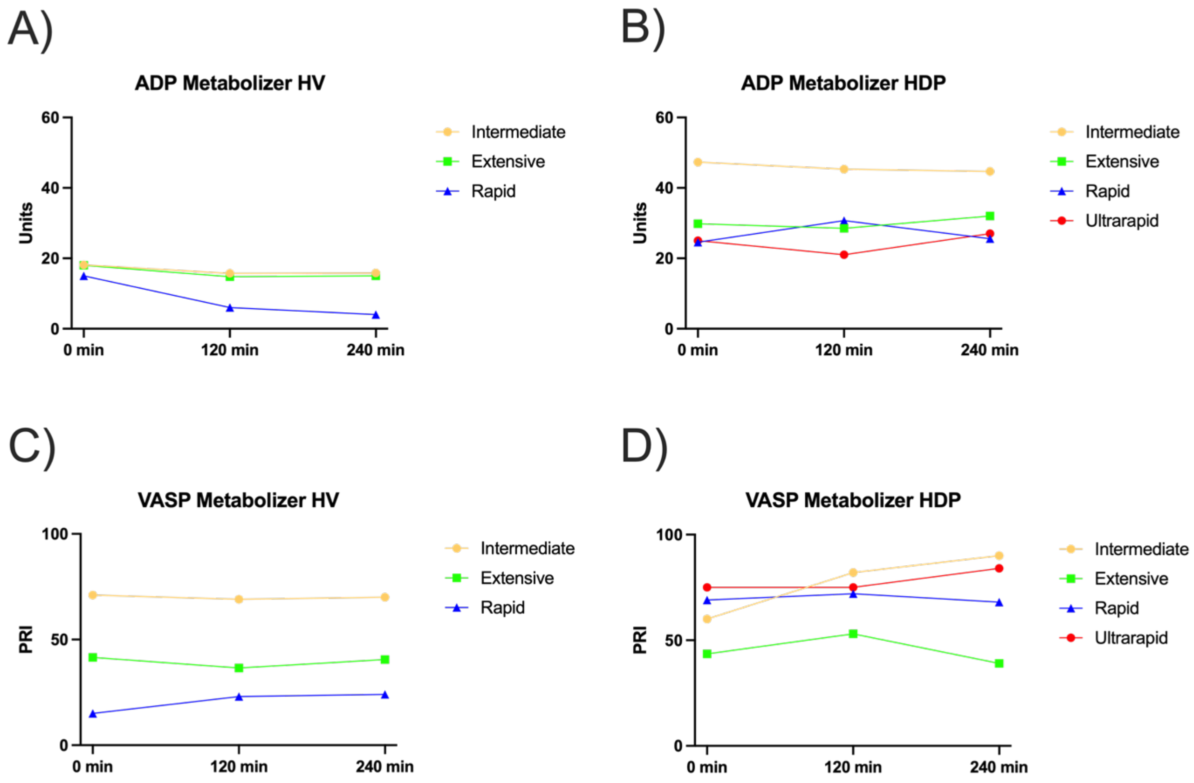

Figure S6. Results grouped by genotypes for healthy subjects (A,C) and hemodialysis patients (B,D). Multiplate (A,B) and VASP (C,D) are presented over time. Abbreviations: ADP: adenosine diphosphate; HV, healthy volunteers; HDP, hemodialysis patients; VASP: vasodilator-stimulated phosphoprotein

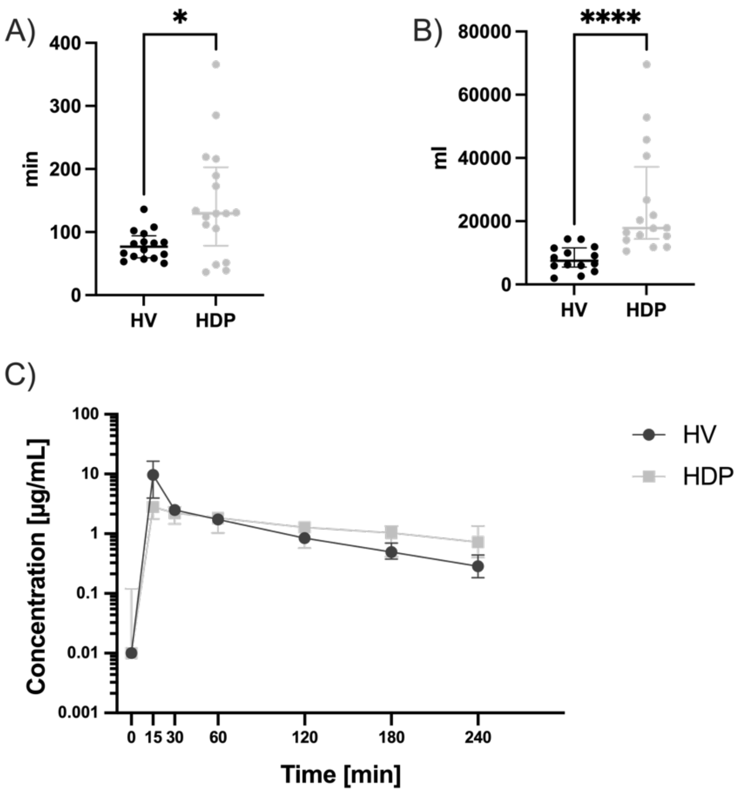

Figure S7. The half-life (A), volume of distribution (b), and concentration of pantoprazole over time (C). HV, Healthy Volunteers; HDP, Hemodialysis Patients

Supplementary References

S1. Jung YS, Jin BH, Park MS, et al. Population pharmacokinetic-pharmacodynamic modeling of clopidogrel for dose regimen optimization based on CYP2C19 phenotypes: A proof of concept study. CPT Pharmacometrics Syst Pharmacol 2024;13(1):29-40

S2. Jiang XL, Samant S, Lewis JP, et al. Development of a physiology-directed population pharmacokinetic and pharmacodynamic model for characterizing the impact of genetic and demographic factors on clopidogrel response in healthy adults. Eur J Pharm Sci 2016;82:64-78

S3. Beal SL. Ways to fit a PK model with some data below the quantification limit. J Pharmacokinet Pharmacodyn 2001;28(5):481-504

Reporting checklist for randomised trials (based on the CONSORT guidelines)

		Reporting Item	Page Number
**Title and Abstract**			
Title	#1a	Identification as a randomized trial in the title.	na
Abstract	#1b	Structured summary of trial design, methods, results, and conclusions	2
**Introduction**			
Background and objectives	#2a	Scientific background and explanation of rationale	4
Background and objectives	#2b	Specific objectives or hypothesis	5
**Methods**			
Trial design	#3a	Description of trial design (such as parallel, factorial) including allocation ratio.	5
Trial design	#3b	Important changes to methods after trial commencement (such as eligibility criteria), with reasons	na
Participants	#4a	Eligibility criteria for participants	6
Participants	#4b	Settings and locations where the data were collected	5
Interventions	#5	The experimental and control interventions for each group with sufficient details to allow replication, including how and when they were actually administered	6

Outcomes	#6a	Completely defined prespecified primary and secondary outcome measures, including how and when they were assessed	8
Sample size	#7a	How sample size was determined.	8
Sample size	#7b	When applicable, explanation of any interim analyses and stopping guidelines	na
Randomization - Sequence generation	#8a	Method used to generate the random allocation sequence.	
na			
Randomization - Sequence generation	#8b	Type of randomization; details of any restriction (such as blocking and block size)	
na			
Randomization - Allocation concealment mechanism	#9	Mechanism used to implement the random allocation sequence (such as sequentially numbered containers), describing any steps taken to conceal the sequence until interventions were assigned	na
Randomization - Implementation	#10	Who generated the allocation sequence, who enrolled participants, and who assigned participants to interventions	na
Blinding	#11a	If done, who was blinded after assignment to interventions (for example, participants, care providers, those assessing outcomes) and how.	na
Blinding	#11b	If relevant, description of the similarity of interventions	na

Statistical methods	#12a	Statistical methods used to compare groups for primary and secondary outcomes	7
Statistical methods	#12b	Methods for additional analyses, such as subgroup analyses and adjusted analyses	8
Outcomes	#6b	Any changes to trial outcomes after the trial commenced, with reasons	na
**Results**			
Participant flow diagram (strongly recommended)	#13a	For each group, the numbers of participants who were randomly assigned, received intended treatment, and were analysed for the primary outcome	10
Participant flow	#13b	For each group, losses and exclusions after randomization, together with reason	10
Recruitment	#14a	Dates defining the periods of recruitment and follow-up	10
Recruitment	#14b	Why the trial ended or was stopped	na
Baseline data	#15	A table showing baseline demographic and clinical characteristics for each group	21
Numbers analysed	#16	For each group, number of participants (denominator) included in each analysis and whether the analysis was by original assigned groups	10
Outcomes and estimation	#17a	For each primary and secondary outcome, results for each group, and the estimated effect size and its precision (such as 95% confidence interval)	10
Outcomes and estimation	#17b	For binary outcomes, presentation of both absolute and relative effect sizes is recommended	na

Ancillary analyses	#18	Results of any other analyses performed, including subgroup analyses and adjusted analyses, distinguishing pre-specified from exploratory	10
Harms	#19	All important harms or unintended effects in each group (For specific guidance see CONSORT for harms)	na
**Discussion**			
Limitations	#20	Trial limitations, addressing sources of potential bias, imprecision, and, if relevant, multiplicity of analyses	16
Interpretation	#22	Interpretation consistent with results, balancing benefits and harms, and considering other relevant evidence	13
Registration	#23	Registration number and name of trial registry	5
Generalisability	#21	Generalisability (external validity, applicability) of the trial findings	16
**Other information**			
Interpretation	#22	Interpretation consistent with results, balancing benefits and harms, and considering other relevant evidence	13
Registration	#23	Registration number and name of trial registry	5
Protocol	#24	Where the full trial protocol can be accessed, if available	na
Funding	#25	Sources of funding and other support (such as supply of drugs), role of funders	17

The CONSORT checklist is distributed under the terms of the Creative Commons Attribution License CC-BY. This checklist was completed on 24. September 2023

using https://www.goodreports.org/, a tool made by the EQUATOR Network in collaboration with Penelope.ai
